# Traffic Air Pollution and Oxidized LDL

**DOI:** 10.1371/journal.pone.0016200

**Published:** 2011-01-19

**Authors:** Lotte Jacobs, Jan Emmerechts, Marc F. Hoylaerts, Chantal Mathieu, Peter H. Hoet, Benoit Nemery, Tim S. Nawrot

**Affiliations:** 1 Occupational and Environmental Medicine, Unit of Lung Toxicology, Katholieke Universiteit Leuven, Leuven, Belgium; 2 Center for Molecular and Vascular Biology, Katholieke Universiteit Leuven, Leuven, Belgium; 3 Department of Endocrinology, Katholieke Universiteit Leuven, Leuven, Belgium; 4 Centre for Environmental Sciences, Hasselt University, Diepenbeek, Belgium; Instituto de Química, Universidade de São Paulo, Brazil

## Abstract

**Background:**

Epidemiologic studies indirectly suggest that air pollution accelerates atherosclerosis. We hypothesized that individual exposure to particulate matter (PM) derived from fossil fuel would correlate with plasma concentrations of oxidized low-density lipoprotein (LDL), taken as a marker of atherosclerosis. We tested this hypothesis in patients with diabetes, who are at high risk for atherosclerosis.

**Methodology/Principal Findings:**

In a cross-sectional study of non-smoking adult outpatients with diabetes we assessed individual chronic exposure to PM by measuring the area occupied by carbon in airway macrophages, collected by sputum induction and by determining the distance from the patient's residence to a major road, through geocoding. These exposure indices were regressed against plasma concentrations of oxidized LDL, von Willebrand factor and plasminogen activator inhibitor 1 (PAI-1). We could assess the carbon load of airway macrophages in 79 subjects (58 percent). Each doubling in the distance of residence from major roads was associated with a 0.027 µm^2^ decrease (95% confidence interval (CI): −0.048 to −0.0051) in the carbon load of airway macrophages. Independently from other covariates, we found that each increase of 0.25 µm^2^ [interquartile range (IQR)] in carbon load was associated with an increase of 7.3 U/L (95% CI: 1.3 to 13.3) in plasma oxidized LDL. Each doubling in distance of residence from major roads was associated with a decrease of −2.9 U/L (95% CI: −5.2 to −0.72) in oxidized LDL. Neither the carbon load of macrophages nor the distance from residence to major roads, were associated with plasma von Willebrand factor or PAI-1.

**Conclusions:**

The observed positive association, in a susceptible group of the general population, between plasma oxidized LDL levels and either the carbon load of airway macrophages or the proximity of the subject's residence to busy roads suggests a proatherogenic effect of traffic air pollution.

## Introduction

Numerous epidemiological studies link various adverse health outcomes with air pollution, especially that caused by particulate matter (PM), which to a considerable extent is caused by traffic [1], [2]. One of the important recent discoveries has been that exposure to PM is not only harmful to the lungs, but also to the heart and blood vessels [3]–[6]. This is undoubtedly true for short-term increases in PM, which are triggers for acute cardiovascular events [7], but probably also for long-lasting exposure to urban PM, which increases the risk of cardiovascular mortality and morbidity [4], [6], possibly by accelerating atherosclerosis [8]–[10]. A cross-sectional study in Los Angeles [9] suggested a role of air pollution in intima-media thickening of the carotid artery and a follow-up study described an association between traffic proximity and the progression of intima-media thickness [10]. In a German study of more than 4000 subjects a strong relation was found between coronary artery calcification and living close to major roads [8]. These epidemiological observations strongly suggest that long-term exposure to PM exerts a proatherogenic effect. Studies in laboratory animals have begun to give experimental plausibility to these epidemiological observations [11], [12]. However, so far, only few studies have provided mechanistic evidence for an effect of chronic exposure to traffic air pollution on the development of atherosclerosis in human subjects.

It is well established that persons with diabetes have a higher risk of developing cardiovascular diseases. A population-based study showed that persons with diabetes, without previous myocardial infarction, have the same risk of developing myocardial infarction as nondiabetic patients with previous myocardial infarction [13]. The metabolic abnormalities caused by diabetes induce vascular dysfunction that predispose these patients to developing atherosclerosis [14]. There is also evidence that persons with diabetes and cardiovascular disease are more sensitive to the effects of PM air pollution [15]. So it is relevant – and also probably easier – to study the effects of air pollution in this more susceptible fraction of the population. Thus, in a previous study in diabetic subjects, we showed associations between recent exposure to PM and systemic inflammation, and between recent PM and platelet activation, indicative of a prothrombotic tendency [16]. A strong point of that study is that we were also able to estimate the participants' exposure to chronic air pollution at the individual level by the carbon load of airway macrophages obtained by induced sputum. The carbon load of airway macrophage reflects a subject's exposure to soot derived from the combustion of fossil fuels, as demonstrated in children [17]. However, we also wanted to test the hypothesis that chronic air pollution would impact on indices or predictors of atherosclerosis. Therefore, we measured the concentration of oxidized LDL in plasma samples from this same population, because oxidized LDL is a well-established biomarker of (subclinical) atherosclerosis and plaque formation [18]. We also measured plasma von Willebrand factor and PAI-1, as markers for endothelial dysfunction [19], [20].

We hypothesized that chronic exposure to PM, as assessed by the carbon load of airway macrophages, was associated with an increase in the concentrations of circulating oxidized LDL, in a presumably more susceptible population.

## Methods

### Participants

The present study population is drawn from the one previously described [16]. Briefly, non-smoking persons with either type 1 or type 2 diabetes were recruited consecutively from the diabetes outpatient clinic at the University Hospital Leuven. Of the 186 recruited subjects, 137 (74%) consented and took part in the examination. Sufficient numbers of airway macrophages, to assess the area occupied by carbon, were obtained from 80 of the 119 patients (18 of the 137 patients failed to produce sputum). Of these 80 subjects with sufficient numbers of airway macrophages, oxidized LDL was available in 79, because in one person a blood sample could not be obtained. Patients completed a questionnaire to obtain information on age, occupation, socioeconomic status, exposure to environmental tobacco smoke, alcohol use, use of medication and place of residence. Socioeconomic status was coded and condensed into a scale with scores ranging from 1 to 3, on the basis of education and occupation of the patients. Determination of underlying cardiovascular diseases was based on the tenth International Classification of Diseases (ICD-10 code: I20-I89).

### Ethics

The Ethics Review Board of the Medical Faculty of the University of Leuven (K.U.Leuven) approved the study. Participants gave written informed consent at recruitment.

### Exposure assessment

#### Carbon load of airway macrophages obtained by induced sputum

**Figure 1:**
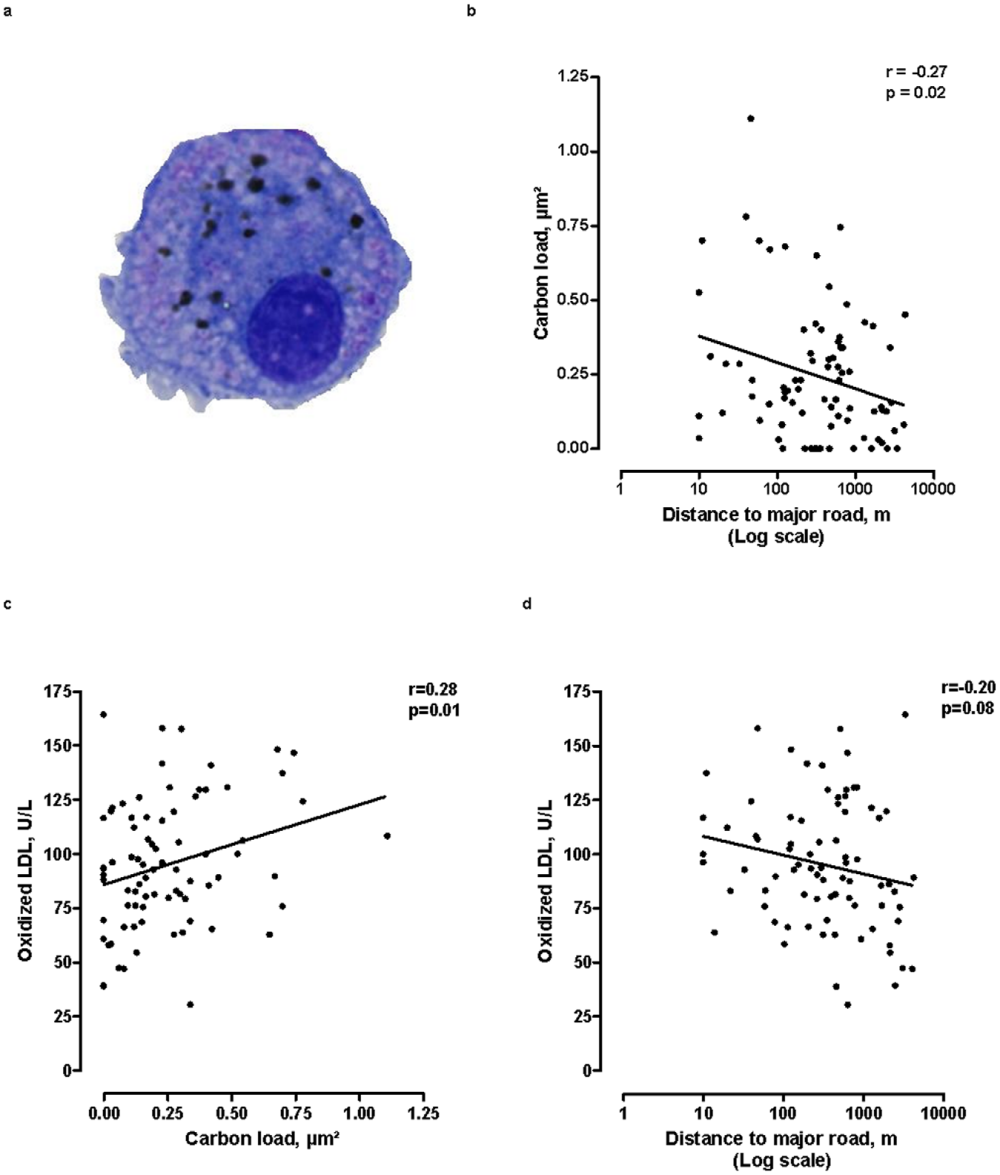
Traffic related exposure variables and oxidized-LDL. An airway macrophage containing carbon particles (A). We determined the surface of the macrophage occupied by carbon (in µm^2^), in 50 macrophages per person. The carbon load is given as the median carbon load of 50 airway macrophages. Pearson correlation between carbon load of airway macrophages and distance from the residence to a major road (B). (Data leading to panel b have been previously published [16]). The shortest distance to a major road is 10 meters, by definition. Pearson correlation between plasma concentrations of oxidized LDL and carbon load of airway macrophages (C) and between plasma concentrations of oxidized LDL and distance from the residence to a major road (D).

The induction of sputum in the patients and the processing of induced sputum was previously described. Briefly, nebulized saline (NaCl 3, 4 or 5%) was administered through an ultrasonic nebulizer (Ultra-NebTm2000 model 200HI, De Vilbiss Healtcare, Somerset, PA, USA) in one, two or three 7-min inhalation periods. To isolate airway macrophages, induced sputum was processed according to a standard technique [21]. Airway macrophages were visualized by light microscopy (AxioPlan 2 Imaging, Zeiss, Zaventem, Belgium). Then Scion image software (Scion Corporation, Frederick, MD, USA) was used to calculate the carbon load of airway macrophages (Figure 1A), which was defined as the median area (µm^2^) occupied by carbon, in 50 randomly selected macrophages per patient [16], [17], [22].

#### Distance to major roads

Distances from the patient's residence to a major road were calculated through geocoding (the shortest distance is 10 meters). A major road was defined as an N-road (major traffic road) or an E-road (motorway/highway) [16].

#### Recent exposure

A portable laser-operated aerosol mass analyzer (Aerocet 531, Met One Instruments Inc, Grant Pass, OR, USA) was used to measure PM_2.5_ (particles with an aerodynamic diameter of less than 2.5 µm) and PM_10_ concentrations two hours before the patient's participation in the study. The device had been previously calibrated against a local monitoring station (Flemish Environmental Agency, Borgerhout, Antwerp). The PM concentrations were measured outside, at the entrance of the hospital, as previously described [16].

### Clinical measurements

#### Blood collection

As previously described [16], non-fasting blood samples were collected in an EDTA tube and on 0.129 M (3.8%) sodium citrate, on the same day as the sputum collection, according to standard clinical procedures. Blood cell counts and differential leukocyte counts were determined using an automated cell counter with flow differential (Cell Dyn 3500, Abbott Diagnostics, Abott Park, IL, USA). Plasma samples were kept frozen at −80°C for future analysis.

#### Biochemical analysis

Oxidized LDL concentration in plasma was measured by a commercially available sandwich ELISA (Mercodia, Uppsala, Sweden). Antigen levels of plasma von Willebrand factor and PAI-1 were measured with an in-house ELISA. Blood glucose levels, glycated haemoglobin, total cholesterol, high-density lipoprotein (HDL) cholesterol and triglycerides were measured according to standard clinical procedures. LDL was calculated from the Friedewald formula [23].

### Statistical analysis

For database management and statistical analysis, we used SAS Software (version 9.1, SAS Institute Inc, Cary, NC). Non-normally distributed data were log transformed. We investigated associations between plasma concentrations of oxidized LDL, von Willebrand factor and PAI-1 and markers of chronic exposure (carbon load of airway macrophages and distance from residence to major roads) using stepwise linear regression in which we set p = 0.15 for the independent variables to enter and to stay in the model. Covariates considered for entry in the model were sex, age, body-mass index (BMI), type of diabetes, socioeconomic status, hour of blood draw, exposure to environmental tobacco smoke, physical activity, LDL, HDL, blood glucose level, glycated haemoglobin, blood leukocyte counts, use of statins, use of angiotensin-converting–enzyme (ACE) inhibitors and use of antiplatelet medication. Irrespective of selection by the stepwise regression model we forced sex, age, body-mass index, socioeconomic status, type of diabetes, glycated haemoglobin levels, statin use and blood leukocyte counts into the models. We ran three models: model 1 unadjusted analysis, model 2 adjusted for sex, age, socioeconomic status, LDL and HDL cholesterol and finally a fully adjusted model 3 for which additional covariates were selected by stepwise regression. We applied multiple logistic regression analysis to study the relation between clinical plasma levels of oxidized LDL [18] and the carbon load of airway macrophages. We defined high plasma oxidized LDL as levels above the 75^th^ percentile (>117 U/L), which corresponds to a higher risk for moderate to large plaques [18]. Potential interactions between carbon load of airway macrophages and type of diabetes, glycated haemoglobin and use of statins on plasma levels of oxidized LDL were investigated. Q-Q plots of the residuals were used to test the assumptions of all linear models.

## Results

### Characteristics of study participants

The present study population is drawn from the one previously described [16]. The characteristics of the 79 patients (age range: 22–78 years) in whom the macrophage carbon load could be determined (58%) are described in Table 1.

**Table 1:** Patient characteristics (n = 79).

	Mean (SD) or number (%)
Women	37 (47%)
Age, years	56.5 (14.3)
BMI, kg/m^2^	28.7 (5.2)
Type 1 diabetes	33 (42%)
Exposure to environmental tobacco smoke	11 (14%)
Socioeconomic status	
Low	44 (56%)
Middle	24 (30%)
High	11 (14%)
Medication use	
Antiplatelet medication	46 (58%)
Statins	48 (61%)
ACE inhibitor	31 (39%)
Insulin	75 (95%)
Oral antidiabetic medication	33 (42%)
Underlying cardiovascular disease	30 (38%)
Blood glucose, mg/dL	147 (68)
Glycated haemoglobin, %	7.4 (1.0)
Total blood leukocytes, /µL	6153 (1996)
Blood platelets, ×10^3^/µL	230 (62)
von Willebrand factor, µg/mL	13.6 (5.3)
PAI-1, ng/mL	84.4 (66.5)
Cholesterol, mg/dL	158 (34)
Triglycerides, mg/dL	132 (66)
LDL, mg/dL	81.0 (26.2)
HDL, mg/dL	50.7 (18.7)
Oxidized LDL, U/L	85.6 (31.4)

### Carbon load of airway macrophages and distance to major road

The median carbon load of our patient's airway macrophages was 0.20 µm^2^ (25^th^–75^th^ percentile: 0.095 to 0.34 µm^2^) and the median distance from the residence to a major road was 400 meters (25^th^–75^th^ percentile: 124 to 839 meters). The relation between carbon load in airway macrophages (expressed as a surface), and distance between residence and major roads is depicted in Figure 1B. Each doubling in distance to major roads was associated with a significant decrease – by 0.027 µm^2^ (95% CI, −0.048 to −0.0051; p = 0.02) – in the carbon load of airway macrophages. These data further validate the use of this novel biomarker – proposed in a study of children [17] – as an indicator of a subject's previous exposure to traffic-related air pollution.

### Determinants of oxidized LDL

In stepwise regression analysis, plasma oxidized LDL concentration was independently and positively correlated with LDL (regression coefficient ± SE, 0.78±0.10 U/L per mg/dL; p<0.001) and inversely correlated with high-density lipoprotein (HDL) cholesterol levels (−0.38±0.14 U/L per mg/dL; p = 0.01). Although sex, age, socioeconomic status, BMI, type of diabetes, glycated haemoglobin levels, statin use and blood leukocyte counts were not significantly associated with oxidized LDL, we forced these variables, together with LDL and HDL cholesterol levels, into the regression models. Both before adjustment (Figure 1C, Table 2) and after adjustment (Table 2) for the aforementioned variables, plasma oxidized LDL concentrations were positively associated with the carbon load of airway macrophages: an interquartile (IQR) increase in carbon load (0.25 µm^2^) was associated with an increase of 7.3 U/L (95% CI: 1.3 to 13.3) in oxidized LDL. Distance from residence to major roads tended to be inversely associated with oxidized LDL (Figure 1D, Table 2). After accounting for sex, age, socioeconomic status, BMI, type of diabetes, glycated haemoglobin levels, statin use, blood leukocyte counts and LDL and HDL cholesterol, each doubling in the distance from the patient's residence to a major road was associated with a decrease of 2.9 U/L (95% CI: −5.2 to −0.72) in plasma levels of oxidized LDL (Table 2).

**Table 2:** Estimated change in plasma oxidized LDL levels in association with carbon load or distance from residence to major roads.

Carbon load*, +0.25 µm^2^			
	Estimate	95% CI	p-value
Model 1	9.3	2.1 to 16.4	0.01
Model 2	6.7	1.2 to 12.2	0.02
Model 3	7.3	1.3 to 13.3	0.02

Distance to major road†, ×2			
	Estimate	95% CI	p-value
Model 1	−2.6	−5.5 to 0.31	0.08
Model 2	−2.9	−5.0 to −0.73	0.01
Model 3	−2.9	−5.2 to −0.72	0.01

Estimates reflect the change in oxidized LDL (U/L); CI = confidence interval.

Model 1: Unadjusted.

Model 2: Adjusted for sex, age, LDL and HDL cholesterol.

Model 3: Adjusted for sex, age, socioeconomic status, LDL and HDL cholesterol, BMI, type of diabetes, glycated haemoglobin, statin use and blood leukocyte counts.

*Effect size calculated for an interquartile range difference in carbon load.

† Effect size was calculated for a twofold increase in distance from residence to major road (based on a model with log distance).

Oxidized LDL was not associated with total blood leukocyte counts, nor with plasma von Willebrand factor or Plasminogen Activator Inhibitor-1 (PAI-1).

### Determinants of von Willebrand factor and PAI-1

Neither the carbon load of macrophages nor the distance from residence to major roads, were associated with plasma von Willebrand factor or PAI-1, taken as indices of endothelial dysfunction.

### Sensitivity analysis

Studies have pointed to reduced susceptibility to the effects of air pollution in those that take statins [24], [25]. We therefore tested the interaction term of carbon load of macrophages by statin use. The interaction term of carbon load and oxidized LDL by statin use tended to be significant (p = 0.09) in models that did not account for LDL and HDL cholesterol but did not reach statistical significance (p = 0.64) in models that did account for plasma cholesterol levels.

There was also no effect-modification by sex (p = 0.51 for interaction), age (p = 0.55) type of diabetes (p = 0.86), percentage glycated haemoglobin (p = 0.47), and BMI (p = 0.65) on the association between carbon load and oxidized LDL. Oxidized LDL did not correlate with recent PM air pollution measured at the hospital on the day of the patient's visit.

## Discussion

The key finding of our study is that plasma oxidized LDL concentration, a molecular marker of subclinical atherosclerosis, is positively associated with the carbon load of airway macrophages, a marker of chronic exposure to carbon particles derived from fossil fuel burning. This association could not be explained by sex, age, socioeconomic status, LDL and HDL cholesterol levels, BMI, type of diabetes, glycated haemoglobin levels, statin use, blood leukocyte counts or any other covariate studied.

Experimental work in animals has already shown associations between exposure to air pollution and oxidized LDL. Mice had increased IgM antibody titres to copper oxidized LDL after five weeks of exposure to cigarette smoke [26]. Exposure to urban air pollution for four months exacerbated the susceptibility of LDL to oxidation in hyperlipemic mice and levels of anti-oxidized LDL antibodies were significantly higher in mice on a high fat diet when exposed to urban air pollution [27].

We found no association between exposure to chronic air pollution and markers of endothelial function (von Willebrand factor and PAI-1), and also no association between these endothelial markers and oxidized LDL was found. Further, oxidized LDL was not associated with blood leukocytes, although we previously showed associations between exposure to air pollution and blood leukocyte counts [16]. This suggests that the mechanism underlying the association between chronic exposure to PM and oxidized LDL is independent of the one underlying the association between air pollution and inflammatory changes, such as increases in blood leukocyte counts. In this context, the oxidative potential of air pollutants can play a role in the observed association, since oxidized LDL has been identified as a marker of oxidative stress [28]. In susceptible apolipoprotein E-deficient mice, concentrated ultrafine particles caused systemic oxidative stress, an inhibition of the anti-inflammatory capacity of HDL, and larger early atherosclerotic lesions [29]. Studies in humans showed associations between plasma homocysteine level and exposure to PM_2.5_ and black carbon [30]. Oxidative modification of LDL, together with increased blood leukocytes and platelets, contributes to the initiation and progression of atherosclerosis [31], [32]. We showed that exposure to particles can have an effect on both these processes. Increased circulating levels of oxidized LDL are associated with adverse cardiovascular outcomes. In a population-based prospective study in 326 healthy men, plasma oxidized LDL levels, measured at baseline, predicted the occurrence and size of atherosclerotic plaques in the carotid arteries, three years later [18]. In our sample, a quarter of the subjects had oxidized LDL concentrations above 117 U/L, a level previously associated with large risk of having carotid plaques [18]. Here, the odds of having plasma oxidized LDL levels above that value increased by 163% for an IQR increase in the carbon load of airway macrophages. Findings from a nested case-control study suggest that a high plasma oxidized LDL/total cholesterol ratio can be a possible indicator of increased risk for acute myocardial infarction [33]. Holvoet et al. [34] also showed that patients with coronary artery disease had higher levels of oxidized LDL compared with age-matched controls without clinical evidence of cardiovascular disease.We did not find evidence of a higher sensitivity to pollution-induced effects on oxidized LDL in persons with type 2 diabetes compared with their type 1 counterparts. However, our patients had well-controlled glycated haemoglobin levels (average 7.4%) and insulin use in persons with type 2 diabetes was high (95%).

Our study has limitations. Observational studies do not prove causality, even when exposure is estimated on an individual level. Determining the carbon load in 50 randomly selected macrophages per patient is a labour-intensive technique and suitable sputum samples cannot always be obtained. Although sputum induction was successful in only 58 percent of our patients, this is unlikely to have introduced bias, since there were no differences between those in whom sputum was induced successfully and those in whom it was not [16]. We also did not observe any significant differences in exposure (carbon load or proximity to major roads) between the different socioeconomic classes. The exposure markers used here, i.e. carbon load and distances from major road, are surrogates for exposure to traffic-related air pollution. Although we focus on PM, we cannot exclude a role of gaseous pollutants associated with traffic air pollution (NO, NO2). Our study was performed on purpose in a presumably more susceptible fraction of the population, i.e.; diabetic subjects, and this means that our conclusions do not necessarily apply to healthy subjects (with or without atherosclerosis). We encourage to verify our findings in larger populations, such as the Multi-Ethnic Study of Atherosclerosis (MESA) [35].

In conclusion, we showed in a susceptible target population that individually assessed chronic exposure to air pollution is associated with plasma levels of oxidized LDL, a marker of early atherosclerosis. Our findings thus add mechanistic plausibility to the hypothesis that air pollution accelerates the development of atherosclerosis.
